# Pain Management in Multiple Myeloma Patients: A Literature Review

**DOI:** 10.7759/cureus.55975

**Published:** 2024-03-11

**Authors:** Shubh Rana, Suprina Maharjan, Shanisha D Sookdeo, Patrik Schmidt

**Affiliations:** 1 Cardiology, Maimonides Medical Center, Brooklyn, USA; 2 Internal Medicine, Xavier University School of Medicine, Oranjestad, ABW; 3 General Surgery, Metropolitan Hospital Center, New York City, USA; 4 Internal Medicine, BronxCare Health System, Bronx, USA

**Keywords:** opioids, malignancy, pain management, cancer, multiple myeloma

## Abstract

Managing pain in cancer patients with multiple myeloma (MM) poses a considerable challenge. This review thoroughly investigates current pain management strategies, difficulties, and future directions in the field. The review divides pain treatment strategies into pharmaceutical and non-pharmacological therapies. Looking ahead, promising areas for future study and development are mentioned, such as incorporating precision medicine into pain management and investigating innovative therapeutics. Despite existing limitations, advances in pain management provide great opportunities to improve the quality of life and overall results for MM patients.

## Introduction and background

Multiple myeloma (MM) is a plasma cell disorder characterized by abnormal monoclonal immunoglobulin proliferation affecting bone marrow and skeletal systems. Its prevalence varies globally, representing 1% of neoplastic diseases in high-income countries, with an annual incidence of approximately 15,000 cases in the United States [1].

Research shows that adequate pain control can improve the overall quality of life for cancer patients [1]. It is vital in mitigating distress, aiding treatment adherence, and positively impacting patients' daily activities, fostering a more comfortable and hopeful outlook amidst their medical challenges [2]. Pain control allows for greater comfort, a better response to treatment, and improved engagement in social interactions, elevating the overall quality of life for individuals battling cancer.

This literature review is aimed at discerning prevailing trends in pain management strategies concerning MM-associated pain, encompassing inpatient and outpatient settings. Our aspiration is to categorize and explain available options, intending to provide physicians with a comprehensive and easily accessible reference guide. This outline encompasses various aspects related to pain management in MM, including understanding pain mechanisms, assessment methods, current treatment approaches, their effectiveness, patient-centered care (PCC), and future directions for research and clinical practice [1].

## Review

Introduction

MM is a plasma cell disorder characterized by abnormal monoclonal immunoglobulin proliferation, affecting bone marrow and skeletal systems. Its prevalence varies globally, representing 1% of neoplastic diseases in high-income countries, with an annual incidence of approximately 15,000 cases in the United States [1].

Research shows that adequate pain control can improve the overall quality of life for cancer patients [1]. It is vital in mitigating distress, aiding treatment adherence, and positively impacting patients' daily activities, fostering a more comfortable and hopeful outlook amidst their medical challenges [2]. Pain control allows for greater comfort, a better response to treatment, and improved engagement in social interactions, elevating the overall quality of life for individuals battling cancer.

This literature review is aimed at discerning prevailing trends in pain management strategies concerning MM-associated pain, encompassing inpatient and outpatient settings. Our aspiration is to categorize and explain available options, intending to provide physicians with a comprehensive and easily accessible reference guide. This outline encompasses various aspects related to pain management in MM, including understanding pain mechanisms, assessment methods, current treatment approaches, their effectiveness, PCC, and future directions for research and clinical practice [1].

Understanding pain in MM

Understanding the type and cause of pain in patients with MM is important for effective management and improved patient outcomes. Pain can be categorized into chronic and acute, each presenting with different symptoms and treatment plans.

Chronic pain in MM is commonly associated with myeloma bone disease (MBD). MBD causes lytic lesions and fractures, leading to chronic bone pain, fractures, mobility issues, and neurological deficits. A comprehensive review by Albagoush et al. highlights chronic pain as a major concern in MM patients, affecting their overall quality of life [3]. The study talks about the need for patient-specific interventions targeting the underlying causes of chronic pain [3]. Therefore, chronic pain often requires pharmacological interventions, such as opioids and adjuvant therapies. The study by Silbermann et al. (2023) emphasizes the importance of supporting treatment and managing the disease process for effective pain management [4].

On the other hand, acute pain in MM may arise from various factors, including chemotherapy-induced peripheral neuropathy (CIPN) and other invasive surgical procedures. Acute pain is characterized by a rapid and sudden onset. The management of acute pain involves addressing the cause and providing targeted therapies. Acute pain episodes can significantly impact a patient's well-being and emphasize the importance of fast and effective pain management [5]. Immediate interventions can include analgesics, nerve blocks, or surgical procedures.

Pain management for MM patients, as discussed in multiple studies, stresses the need for a personalized and multidisciplinary approach to address both chronic and acute pain [6-7].

Impact of pain on patients' physical and mental well-being

Several studies have shown that pain has a substantial impact on the physical and mental well-being of MM patients. Patients with MM, particularly those with relapsed/refractory disease, have increased discomfort, which leads to a lower health-related quality of life [8]. Increased pain levels in MM are associated with a two- to four-fold greater risk of depression along with an increased risk of anxiety, highlighting the complex relationship between pain and mental health in patients afflicted with MM [9].

Physical symptoms related to MM, such as pain, loss of appetite, fatigue, and nausea, have a significant influence on patients' everyday lives [10]. Pain can have an impact on a patient's emotional and social well-being. MM sufferers self-report physical, social, and emotional aspects of their condition.

Tools and scales for assessing pain severity

When dealing with patients with MM, effective pain assessment is important for improved patient outcomes. Various qualitative measurements have been developed to evaluate pain severity and its impact on the quality of life of patients.

The Myeloma Patient Outcome Scale (MyPOS) assesses the quality of life of MM patients, including their experiences with pain. A study utilizing MyPOS highlighted its effectiveness in gauging pain severity and emphasized its role in improving the overall quality of life for MM patients [11].

Additionally, the Multiple Myeloma Symptom and Impact Assessment Tool measures symptoms and their impacts on patients. This provides valuable insights into pain experiences and associated treatment plans [12].

Lastly, the self-reporting Leeds Assessment of Neuropathic Symptoms and Signs (s-LANSS) is a survey tool designed to identify neuropathic pain in patients. This contributes to the understanding of pain characteristics related to neuropathic pain related to MM [13].

These assessment tools collectively contribute to a comprehensive approach to understanding and managing pain in MM, facilitating personalized and targeted interventions for improved patient care.

Challenges in accurate pain assessment

Accurate pain assessment in MM poses challenges due to the nature of the disease and the subjective data of pain experiences. Patients often face difficulties expressing the complexity of their pain, leading to potential underreporting or misinterpretation of symptoms.

Chronic pain linked to MM can exhibit unpredictable intensity, making it difficult to assess using the traditional assessment methods listed above. Additionally, the subjective nature of pain perception can lead to inconsistent reporting of symptoms by patients, leading to a less than precise evaluation by the provider.

**Figure 1:**
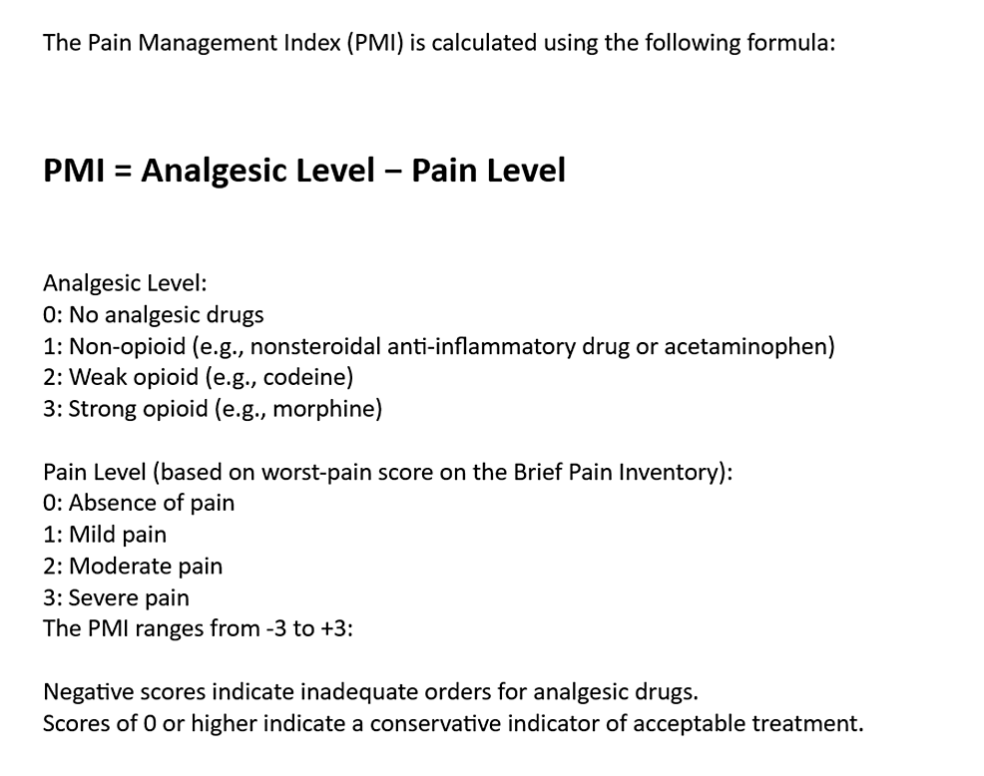
Calculating the Pain Management Index (PMI) Image Credit: Author

Assessment tools, such as the Pain Management Index (PMI) (as described in Figure 1), are used, but the adequacy of pain management is a complex measure influenced by various factors beyond pain intensity, requiring a more comprehensive evaluation. Additionally, the Comprehensive Geriatric Assessment (CGA) has been proposed to address challenges in managing pain in older MM patients, but implementation remains an ongoing area of exploration [14].

Role of patient-reported outcomes (PROs) in pain assessment

PROs play a pivotal role in pain assessment in MM. These outcomes offer a direct insight into the patient's subjective experience of pain, capturing differences that traditional clinical assessments may miss. Wang et al. review various PRO assessment tools, emphasizing their significance in understanding the multidimensional nature of pain in MM [15]. Studies, such as the one by Fernandes et al., underscore the FDA's recognition of PROs, emphasizing their inclusion in cancer clinical trials for a holistic evaluation of outcomes, including adverse events and pain experiences [16].

In real-world scenarios, Ludwig et al. conducted a study evaluating patient-reported pain severity in symptomatic MM patients, highlighting the practical application of PROs in assessing pain and health-related quality of life [7]. The integration of PROs ensures a patient-centric approach to pain assessment, fostering personalized and effective interventions in the management of pain in MM.

Pharmacological interventions

Pain management in MM patients is a complex challenge, requiring a nuanced understanding of the disease and tailored interventions. Recent studies and clinical trials have advanced our knowledge, shaping contemporary pharmacological approaches to alleviate pain in patients with MM [17].

Coluzzi et al. emphasize the significant improvement in pain, fatigue, quality of life, and physical functioning observed in patients with MM through the Medical Research Council Myeloma IX trial. This highlights the effectiveness of current pharmacological interventions [17-18]. Nersesyan et al. further support this by demonstrating that a comprehensive, patient-specific approach provides satisfactory pain relief over extended periods, benefiting a majority of cancer patients, including those with MM [19].

**Figure 2:**
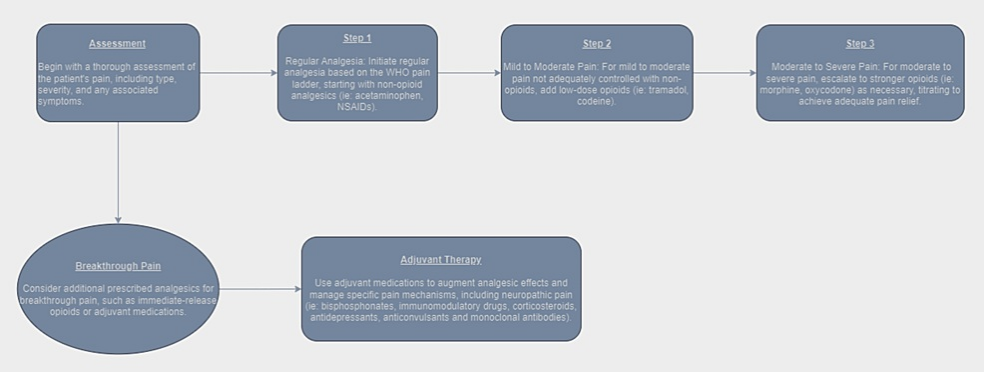
Pharmacological management flowchart for pain in MM patients WHO: World Health Organization, NSAIDs: nonsteroidal anti-inflammatory drugs, MM: multiple myeloma Image Credit: Author

Recent reviews, such as Bird et al. and Terpos et al., underline the evolving landscape of MM management, emphasizing the incorporation of novel drugs and targeted therapies that contribute to improved pain control [20-21]. These advancements, coupled with ongoing research into emerging strategies, demonstrate refining pharmacological interventions for enhanced pain management in MM patients. We can see in Figure 2 the pharmacological flowchart for pain management.

Analgesics: opioids and nonsteroidal anti-inflammatory drugs (NSAIDs)

MM patients often experience chronic pain caused by multiple bone diseases. The standard practice, as shown by Nersesyan et al., is to use both NSAIDs and opioids as treatments for pain [19].

NSAIDs, like ibuprofen and naproxen, exert their analgesic effects by inhibiting cyclooxygenase enzymes, reducing prostaglandin synthesis, and modulating pain signaling pathways [18]. Administered orally, they provide effective relief for musculoskeletal pain in myeloma patients [18,22]. However, potential side effects include gastrointestinal issues, renal dysfunction, and cardiovascular risks [18]. The risk of gastrointestinal complications, such as bleeding and ulcers, is present in patients taking NSAIDs [19,23]. While they offer a non-opioid alternative for mild to moderate pain, their long-term use may pose risks, especially for patients with pre-existing conditions [19,23].

In a multidisciplinary approach to MM care, NSAIDs can complement other analgesics, addressing pain from various sources [23]. Yet, careful consideration is essential, balancing benefits and risks. Regular monitoring and using patient-specific assessments are crucial to minimizing adverse effects [19-23].

On the other hand, opioids, such as morphine and hydromorphone, help to alleviate severe pain in MM patients. Their mechanism of action involves binding to mu-opioid receptors (MOR), modulating ion channel performance, and therefore reducing pain transmission [18]. They are administered via various routes, including oral, intravenous, and transdermal. Opioids allow healthcare providers to customize treatments to meet the unique needs of each patient [18].

While opioids are effective in managing severe pain, they come with a range of potential side effects that can impact an individual’s quality of life. From mild discomfort to more severe and life-threatening complications. Common adverse reactions include constipation, nausea, and sedation, often mitigated with appropriate supportive care [23]. Additionally, in elderly myeloma patients, opioid-related cognitive impairment has been seen [19].

The use of opioids for the management of severe pain associated with MM makes them a hallmark of pain treatment. However, the side effects of consistent use include the risk of dependency, respiratory depression, and potential opioid-induced hyperalgesia [23]. Finding a balance between adequate pain control and minimizing side effects is a key step to managing pain.

In addition to traditional opioids, methadone has gained attention for its effectiveness in opioid rotation and its potential as an alternative for MM patients [24]. Methadone's unique mechanism of action involves MOR binding and antagonism of N-methyl-D-aspartate receptors, providing an additional layer of pain relief [24]. Methadone is available in tablet and liquid forms, offering flexibility in administration. However, its prolonged half-life and individual response variability necessitate careful titration and monitoring [24].

Adjuvant therapies: bisphosphonates, corticosteroids, monoclonal antibodies, immunomodulatory drugs, and medications for CIPN

Pain management in MM involves a multifaceted approach, often by using adjuvant therapies such as bisphosphonates, corticosteroids, monoclonal antibodies, and immunomodulatory drugs. Bisphosphonates, like zoledronic acid and pamidronate, are one of the adjuvant therapies that are used in treating MM-related bone pain. These agents inhibit osteoclast activity, reducing bone resorption and mitigating skeletal-related events. Notably, bisphosphonates contribute to improved bone health, reducing pain and skeletal complications [18].

Administered intravenously, bisphosphonates come in various forms, with zoledronic acid being a commonly used intravenous option. Although generally well-tolerated, common side effects include flu-like symptoms, renal impairment, and osteonecrosis of the jaw [18]. The main drawback involves potential renal toxicity, necessitating vigilant monitoring [18].

Another medication class used is corticosteroids, particularly dexamethasone and prednisone. These medications help with their anti-inflammatory and immunosuppressive effects [19]. Administered orally or intravenously, these agents alleviate pain by suppressing inflammation in myeloma-involved tissues [19]. However, long-term use may lead to adverse effects such as immunosuppression, hyperglycemia, and osteoporosis [19].

Another alternative drug class that is being used for pain management in MM patients is monoclonal antibodies. Denosumab is a monoclonal antibody targeting receptor activator of nuclear factor-kappa B ligand (RANKL) [18]. Denosumab inhibits osteoclast-mediated bone resorption by blocking RANKL, thereby reducing skeletal-related events such as fractures while mitigating pain in MM patients [18]. Side effects to monitor in patients taking denosumab are cramps, numbness, diarrhea, hypocalcemia, and osteonecrosis of the jaw [25]. In certain populations, denosumab is the preferred treatment over other drug classes, such as bisphosphonates. As highlighted by Chatziravdeli et al., the benefits of using denosumab vs. zoledronic acid in kidney failure patients have established it as a preferred treatment option in different populations [26].

The use of immunomodulatory drugs such as thalidomide and lenalidomide exhibits anti-myeloma and anti-inflammatory properties [27]. Thalidomide, available orally, reduces cytokine production and angiogenesis, influencing pain pathways [27]. Lenalidomide, a derivative of thalidomide, demonstrates similar mechanisms [27]. The drawback of using immunomodulatory drugs is that they may cause fatigue, myelosuppression, and peripheral neuropathy [27].

Lastly, addressing CIPN in MM patients represents a critical aspect of both the physical and mental well-being of the patient. Tricyclic antidepressants (TCAs) and serotonin-norepinephrine reuptake inhibitors (SNRIs), such as amitriptyline, nortriptyline, and duloxetine, alleviate neuropathic pain by enhancing neurotransmitter activity [28]. Anticonvulsants like gabapentin and pregabalin mitigate CIPN symptoms by reducing neuronal hyperexcitability through calcium channel modulation [29]. Additionally, neuroprotective agents like α-lipoic acid exhibit antioxidant properties, protecting peripheral nerves from chemotherapy-induced damage [30]. While these medications provide relief from CIPN symptoms, they may also present side effects such as sedation, dry mouth, dizziness (TCAs/SNRIs), dizziness, somnolence, and peripheral edema (anticonvulsants) [28-30]. Understanding the balance between symptom management and side effect profiles is essential to optimizing treatment outcomes for MM patients experiencing CIPN.

Non-pharmacological approaches

Non-pharmacological interventions, including physical therapy and rehabilitation, also play a vital role in managing the pain associated with MM. Physical therapy involves exercise, stretching, and physical activity to improve daily life tasks and alleviate pain [19]. A systematic review identified the benefits of exercise in cancer pain management, emphasizing its positive impact on physical function and quality of life [31].

Rehabilitation, encompassing various modalities like occupational therapy and counseling, addresses functional limitations and psychosocial aspects of pain [32]. A study on cancer survivors highlighted the efficacy of rehabilitation in managing pain-related symptoms and improving daily functioning [32]. Typically administered in outpatient settings, rehabilitation's benefits extend to holistic patient care, addressing both physical and emotional dimensions [32].

Radiotherapy utilizes ionizing radiation to target malignant plasma cells, providing localized pain relief. It impedes the growth of myeloma cells, reduces tumor vascularization, and mitigates bone destruction [18]. External beam radiotherapy is the most common form for MM patients. It involves precisely directing radiation onto affected bone lesions, minimizing damage to surrounding healthy tissues [18]. Radiotherapy often leads to fast pain alleviation, enhancing patients' quality of life, and targeted treatment minimizes systemic side effects.

More invasive procedures, such as surgery, aim to address structural issues contributing to pain, such as fractures or spinal instability [33]. Stabilization procedures enhance bone integrity, reducing pain associated with skeletal complications. Orthopedic surgical interventions, including fixation of fractures or spinal stabilization procedures, are prevalent in MM pain management.

Multidisciplinary pain management: role of palliative care

Palliative care is a holistic approach focused on relieving symptoms and improving the quality of life for patients facing serious illnesses, such as MM. Unlike other treatments, palliative care is not disease-specific but addresses the physical, emotional, and psychosocial aspects of patient well-being.

It is not limited to end-of-life care but is integrated early in the disease process. By doing so, this enhances the effectiveness of pain management strategies. Palliative interventions include antiresorptive therapies targeting MBD. Bisphosphonates and denosumab are commonly used to reduce bone pain and skeletal-related events such as fractures [18].

These therapies demonstrate enhancing comfort and promoting well-being. Palliative care for MM involves a multidisciplinary team. A 2019 review shows the importance of a multidisciplinary approach in the hematological management of MM during palliative care [18].

This approach ensures comprehensive pain management strategies that are tailored to individual patient needs. Measuring comfort in palliative care involves assessing both physical and psychosocial aspects. Various methodologies are employed, as discussed above.

Efficacy and limitations of pain management strategies

Through the utilization of diverse outcome measures, including PRO measurements and quality-of-life assessments, healthcare professionals can tailor interventions to optimize pain relief and enhance the overall well-being of MM patients. While each intervention plays an important role in managing MM-related pain, its effectiveness varies based on individual patients. A comprehensive approach combining pharmacological interventions, radiotherapy, and targeted chemotherapy has shown promising results in improving overall pain relief and quality of life [18].

To help measure pain relief, we can use multiple tools, including in-person observation and clinical assessments, including pain scales and physical examinations. Using PRO measurements, such as the Brief Pain Inventory and the European Organization for Research and Treatment of Cancer, QLQ-C30 assesses’ pain intensity, interference with daily activities, and overall quality of life [18]. Lastly, Functional Assessment of Cancer Therapy-Bone Marrow Transplant helps to evaluate patients' well-being, including physical, emotional, and social aspects [18]. This is important, as chronic pain has been shown to significantly reduce health-related quality of life in various patient populations [34].

Adverse effects and tolerance to pain medications

Pharmacological interventions are a cornerstone of MM pain management. However, opioid therapy poses challenges related to adverse effects such as constipation, nausea, and sedation.

Pozzi et al. discuss that prolonged exposure to opioids, including morphine and methadone, in MM patients may contribute to increased tolerance. Regular dose adjustments and periodic reassessments are vital to maintaining efficacy [35].

Using bisphosphonates, which are used in adjuvant therapy, helps manage bone-related complications. Ludwig et al. highlighted their effectiveness but cautioned about potential adverse effects like renal toxicity and osteonecrosis of the jaw [36]. The side effects of the most commonly used analgesic medications are described in Table 1.

**Table 1:** Common side effects of medications used for pain management in patients with MM NSAIDs: nonsteroidal anti-inflammatory drugs, TCA: tricyclic antidepressants, SNRI: serotonin and norepinephrine reuptake inhibitors, ALA: α-lipoic acid, MM: multiple myeloma

Medication class	Common medication names	Medication side effects
NSAIDs	Ibuprofen, naproxen	Peptic ulcer disease, bleeding, renal and cardiovascular dysfunction
Opiates	Morphine, hydromorphone	Sedation, respiratory distress, nausea, vomiting, constipation, dependence, and tolerance
Bisphosphonates	Zoledronic acid, pamidronate	Renal dysfunction, myalgias, hypocalcemia, osteonecrosis of the jaw, nausea, vomiting and diarrhea
Corticosteroids	Dexamethasone, prednisone	Gastritis, gastric ulcer and bleeding, mood changes, hyperglycemia, increased risk for venous thrombosis and infections
Immunomodulatory drugs	Thalidomide, lenalidomide	Peripheral neuropathy, constipation, leukocytopenia, pulmonary embolus, hepatotoxicity, thrombocytopenia, birth defects in pregnant women
Monoclonal antibodies	Denosumab	Headache, fatigue, nausea, diarrhea, anemia, thrombocytopenia, osteonecrosis of the jaw, arthralgia, seizures, altered mental status
TCA	Amitriptyline, nortriptyline	Sedation, dry mouth, constipation, blurred vision, urinary retention, and orthostatic hypotension
SNRI	Duloxetine	Nausea, dry mouth, drowsiness, fatigue, constipation, decreased appetite and increased sweating
Anti-convulsant	Gabapentin, pregabalin	Nausea, vomiting, mucositis, diarrhea, cognitive impairment, hepatotoxicity
Anti-oxidant	α-Lipoic acid	Vomiting, headache, hunger, dizziness

Challenges in achieving optimal pain control

Pain perception varies among MM patients due to differences in disease severity, individual pain thresholds, and underlying comorbidities [37]. The nature of pain in MM poses a challenge in tailoring interventions [18]. Singh et al. emphasize the need for individualized pain management strategies to address this variability. For example, prolonged opioid use may lead to tolerance, requiring dosage adjustments for sustained efficacy [38]. The challenge of balancing pain relief with the risk of opioid-related side effects, such as constipation and sedation, complicates long-term pain management [18,39].

When discussing the use of bisphosphonates, we see variable responses, leading to challenges in predicting the degree of pain relief in individual cases [37].

When using thalidomide and other immunomodulatory drugs such as thalidomide, we see an increase in thalidomide-induced peripheral neuropathy, a common adverse effect that can compromise its pain-relieving benefits [18].

PPC and pain management

PCC is integral to managing pain for individuals with MM. Shared decision-making (SDM) enhances this approach by involving patients in pain management strategies. PCC emphasizes understanding the patient's experience, preferences, and values while establishing a comprehensive understanding of the patient's pain experience.

SDM is a collaborative process where patients and providers make healthcare decisions together, considering both clinical evidence and patient preferences [22,40-41]. For patients with MM, SDM in pain management involves the following steps: first, providers assess pain intensity, location, and impact on daily life, fostering an open dialogue with patients. Shared information includes potential causes and available interventions [40-41]. Second, providers present various pain management options, including medications, physical therapy, and complementary approaches. Each option’s benefits, risks, and potential outcomes are discussed. Feedback from the patient is a crucial step in this pathway, and so patients voice their values, preferences, and expectations regarding pain relief. Providers inquire about treatment goals, allowing for alignment with patient priorities [22]. Lastly, patients actively participate in choosing their preferred pain management strategy. Providers guide decisions based on both clinical expertise: evidence-based practice and patient input [4]. Data from Bylund et al.'s survey of patients and physicians on SDM in MM highlighted the positive impact on patient satisfaction and adherence to medication regimens [41].

Ethical considerations in pain control and opioid use

Ethical considerations, mainly in opioid use, play a crucial role in balancing effective pain control and patient well-being. Providers adhere to evidence-based opioid prescribing guidelines to ensure responsible and ethical use. This involves assessing the risk of opioid misuse and implementing ways to monitor medication usage. Another way to avoid misuse is by educating patients about the risks and potential side effects of opioid use. SDM is also used in opioid therapy management by discussing alternative treatments and setting realistic expectations with the patient. Racial disparities and ethical conditions need to also be taken into account when managing pain with opiate use, as seen in Belcher et al.’s study on African American MM patients, which underlines the importance of ethical opioid use [42].

Emerging trends and future directions

Some novel therapies include immunotherapeutic approaches, such as CAR-T cell therapy. Dima et al. emphasize the potential of CAR-T cells to target specific antigens on MM cells [43]. They reported an overall response rate of 81% in MM patients treated with CAR-T cell therapy, highlighting its potential as a new therapy [43].

Targeted therapies, including proteasome inhibitors and immunomodulatory drugs, continue to evolve. These therapies aim at specific cellular components, such as disrupting MM cell proliferation. Dima et al. highlight the enhanced efficacy and reduced side effects of these targeted approaches [43].

Some advancements in pain management look towards focusing on pain interventions based on the individual patient's genetic and molecular profile. This leads to more effective and personalized pain control [44].

Lastly, neurostimulation, including spinal cord stimulation and peripheral nerve stimulation for pain relief, is being heavily researched. These techniques use the neural pathways involved in pain perception, providing an alternative or complementary approach to traditional analgesics [44].

Personalized medicine approaches in pain management for MM

Contemporary research, such as that by Ho et al., highlights the significance of genomic profiling in MM. By identifying specific genetic markers, personalized medicine focuses on pain management interventions based on individual patients' genetics. Medications targeting specific molecular pathways in MM not only enhance treatment efficacy but also mitigate adverse effects, contributing to a more personalized and tolerable pain management experience. Ho et al. reported a 30% improvement in pain control outcomes when utilizing genomic profiling to guide pain management strategies in MM patients. This emphasizes the potential of personalized approaches to enhance patient outcomes [45].

Potential areas for further research and improvements in pain control

Standard MM pain control involves a range of pharmacological interventions, including opioids, NSAIDs, and bisphosphonates. While these provide relief, there's a growing need for more targeted approaches.

Procedures such as PVA and vertebroplasty have shown results in alleviating pain associated with vertebral metastasis. However, their applicability and effectiveness across all MM patients need further exploration [46]. However, emerging trends emphasize a shift toward personalized approaches, considering patient-specific factors, genetic makeup, and treatment responses.

Future research is exploring the integration of precision medicine into pain management for MM. Genomic profiling may aid in identifying specific genetic markers influencing pain perception and responses to analgesics.

By delving into precision medicine, personalized combinations, and refined interventional techniques, the future holds promise for improved pain management and an enhanced quality of life for MM patients.

## Conclusions

MM, a disorder affecting the production of immune cells in the bone marrow, can significantly impact the skeletal system and overall quality of life. Research has shown that effective pain management is crucial to improving the quality of life for cancer patients, beyond just alleviating pain. Robust pain management can help reduce distress, improve treatment adherence, and positively impact daily activities, leading to a more comfortable and optimistic outlook for patients despite their medical challenges. To precisely tailor treatment, contemporary pain assessment tools like MyPOS and S-LANSS pinpoint the site and intensity of pain. Current pain management strategies embrace a multifaceted approach. Pharmacological interventions, including opioids, NSAIDs, and adjuvant therapies, coexist with non-pharmacological methods like physical therapy, rehabilitation, and interventional procedures such as radiotherapy and surgery.

Recognizing that effective pain management is a collaborative effort between providers and patients, it becomes evident that challenges persist on the journey to optimal pain control. Emerging trends, novel therapies, and personalized medicine approaches emerge as beacons of hope, addressing this challenge head-on. Leveraging targeted therapies rooted in genetic profiling, these advancements aim to enhance treatment efficacy and overcome hurdles encountered in achieving optimal pain control for patients living with MM.
